# Promoting Healthier Drinking Habits: Using Sound to Encourage the Choice for Non-Alcoholic Beers in E-Commerce

**DOI:** 10.3390/foods10092063

**Published:** 2021-09-01

**Authors:** Brayan Rodríguez, Christian Arroyo, Luis H. Reyes, Felipe Reinoso-Carvalho

**Affiliations:** 1 School of Engineering, Universidad del Valle, Cali 760001, Colombia; brayan.rodriguez.rivera@correounivalle.edu.co; 2 School of Engineering, Universidad de los Andes, Bogotá 111711, Colombia; 3 School of Marketing and Innovation, Muma College of Business, University of South Florida, Tampa, FL 33620, USA; arroyomera@usf.edu; 4 Grupo de Diseño de Productos y Procesos, Department of Chemical and Food Engineering, Universidad de los Andes, Bogotá 111711, Colombia; lh.reyes@uniandes.edu.co; 5 School of Management, Universidad de los Andes, Bogotá 111711, Colombia

**Keywords:** beer, e-commerce, food marketing, multisensory experiences, non-alcoholic beer, sound, expectations

## Abstract

Important institutions, such as the World Health Organization, recommend reducing alcohol consumption by encouraging healthier drinking habits. This could be achieved, for example, by employing more effective promotion of non-alcoholic beverages. For such purposes, in this study, we assessed the role of experiential beer packaging sounds during the e-commerce experience of a non-alcoholic beer (NAB). Here, we designed two experiments. Experiment 1 evaluated the influence of different experiential beer packaging sounds on consumers’ general emotions and sensory expectations. Experiment 2 assessed how the sounds that evoked more positive results in Experiment 1 would influence emotions and sensory expectations related to a NAB digital image. The obtained results revealed that a beer bottle pouring sound helped suppress some of the negativity that is commonly associated with the experience of a NAB. Based on such findings, brands and organizations interested in more effectively promoting NAB may feel encouraged to involve beer packaging sounds as part of their virtual shopping environments.

## 1. Introduction

With beer being one of the most popular drinks [1] and consumers (particularly the youngest) demanding healthier beverage alternatives to moderate their alcohol intake, the sales of non-alcoholic beer (NAB) have steadily risen in recent years [2]. The global NAB market is predicted to grow by around 24% yearly by the end of 2021. Such a market is expected to have an accumulated value greater than US $29 billion by 2026 [3], showing its strategic and progressive importance in the beverages sector. Such increase in value is, among other things, due to the growing awareness of consumers when it comes to the health risks associated with alcohol consumption. For example, in 2019, it was reported that 47% of consumers limited their alcohol consumption compared to 12 months earlier [4]. However, and even though NAB consumption is growing fast compared to alcoholic beers, NAB market share is still negligible [5,6,7].

The World Health Organization (WHO) encourages a shift in consumer attitudes by thoroughly and widely exposing the harms of alcohol consumption abuse. Such abuse causes approximately 3 million deaths each year worldwide, while also contributing to diverse disabilities and poor health of millions of consumers. Overall, the abuse of alcohol consumption is responsible for more than 5% of the global burden of disease [8]. Consequently, the WHO is somehow calling attention to the growing need to reduce alcoholic drinks’ consumption. Since beer is consistently one of the most consumed beverages globally, nudging consumers to choose the alcohol-free version of a beer while shopping may be an effective way to reduce alcohol consumption. In this sense, a NAB is produced with the same raw materials as an alcoholic beer, which provides a range of similar experiential sensory cues that may be useful to prime the consumer during beer-related decision-making tasks [9]. Hence, to improve the acceptability of NAB while fomenting healthier drinking habits, in this study, we proposed to nudge consumers towards more effectively choosing NAB while shopping for drinks. For this, we focused on modulating their emotions and sensory expectations associated with a NAB via digital and multisensory customization.

### Theoretical Framework

There is scarce research on which variables influence NAB emotions and sensory expectations. For instance, evidence suggests that a NAB is usually perceived as a functional substitute for alcoholic beverages and not a hedonic one [10]. The latter study showed that while being consumed, a NAB mostly evoked neutral or negative emotional responses compared to the emotions elicited by an alcoholic beer or a wine. Meanwhile, in 2017, Silva et al. [11] conducted another study on how two product names, “beer” and “NAB,” might affect such products’ conceptualizations and, thus, consumers’ responses. Here, participants consumed labeled alcoholic beer and NAB in a café/bar, and in some cases, these drinks were mislabeled. The obtained results indicated that the label “NAB” itself was systematically associated with negative emotions (sadness, disappointment, irritability). On the contrary, positive emotions, such as amused, joyful, and excited, were commonly associated with the product labeled “beer.” In other words, both studies mentioned above revealed that a NAB might constantly be framed as a product associated with negativity by consumers. In fact, according to Blackmore et al. [12], non-alcohol and low-alcohol content on beer labels tend to be associated with sensory expectations related to negative beer tasting experiences, such as an excessive sweetness, lack of bitterness, and a light body.

Note that the studies mentioned earlier only focused on the influence of visual and/or flavor sensory cues. However, consumer perception has been extensively discussed as inherently multisensorial [13], where sounds could play a crucial role in consumption decision-making tasks. Although product design and multisensory marketing are growing research fields [14], to the best of our knowledge, there is little research on the role of sonic cues on expectations and/or emotions related to NAB experiences.

Packaging sounds are more and more considered as added-value during a purchase experience. Several brands increasingly use sounds to distinguish themselves from the competition while communicating functional expectations about products [15]. Indeed, people can discern the viscosity, carbonation, and temperature of a beverage just by hearing its pouring sound [16]. Therefore, producers of healthy and dietetic foods could also consider using sounds to more effectively promote this kind of value proposition associated with their products, such as lower alcohol/calories/fat/sugar/salt. In line with the above, we decided to encourage NAB consumption by employing experiential beer packaging sounds in a multisensory shopping context.

A very recent study employed experiential beer packaging sounds to assess beer “premiumness” [17]. Here, the participants listened to opening and pouring beer sounds from a bottle and a can at different sound pressure levels and frequency ranges. They found evidence that the participants perceived the opening sounds of bottles as more premium than those of cans, and the pouring sounds as more premium than the opening ones. Moreover, they also found that the louder opening sounds were perceived as more premium than the quieter ones. This recent study also showed that the more a sound was associated with a premium beer, the more alive, good, pleasant, and happy the participants evaluated it. As a complement, and concerning hedonic sensations associated with high audible frequency ranges (i.e., pitch), another study also reported that high-pitched product opening sounds were more associated with somewhat positive concepts, such as brisk, comfortable, and secure [18].

Considering the aforementioned, and together with the fact that customers spend increasing amounts of time shopping and researching for products online [19], it may be useful for retailers to rely on multisensory product cues as part of digital marketplace experiences, such as packaging sounds. Hence, we decided to frame this new study within the scope of e-commerce experiences. In this context, Ringler et al. [20] recently explored how the sounds generated by the regular operation of a product, while coupled with corresponding imagery (in a screen or utilizing a virtual reality experience), would encourage customers to focus on the product in use and ultimately enhance their willingness to pay. In such a context, they found that louder sounds (vs. quieter) positively affected the perceived product’s power and improved the willingness to pay for it.

Sounds are undoubtedly rich in information while evoking product expectations and emotional responses towards products. However, their use in digital retailing spaces remains limited, highlighting a disconnection between marketing theorists and practitioners [21,22]. Hence, we believe that more research is needed on the role of sound on consumers’ behavior in e-commerce experiences. It is acknowledged that sounds placed on e-commerce create positive feedback from consumers [23]. For instance, research suggests that by using music as an interactive element displayed on e-commerce, consumers tend to evaluate better the purchase experience while increasing their cognitive involvement with the purchase, and consequently, increasing their purchase intention [24]. Therefore, some brands use sounds as a strategy to engage consumers. An example of this is the case of the Lays brand, which consistently uses sounds in their digital interaction with consumers to encourage potato chips’ consumption by making consumers listen to crunchy sounds while purchasing their products [25].

Notably, previous research has suggested that emotions are also a critical component of a product’s user experience [26], and where sounds can play a crucial role as they can activate emotional responses through basic concept associations [27]. The sounds of beverage manipulation, for example, have been found to be more pleasant than other product sounds [28]. Previous evidence has also shown the influence that packaging sounds can prompt on sensory expectations or actual taste perceptions [29,30,31]. Accordingly, we addressed the present study focusing on emotions and sensory expectations. Importantly, multisensory approaches in online retail experiences are becoming increasingly relevant as part of food and beverage marketing strategies as well. Hence, researchers may be encouraged to look for new and creative ways to implement effective cues across the senses to meet the growing online shopping demand [20,32].

To summarize, the main objective of this study was to find out how intrinsic product sounds nudge the purchase of a NAB in digital contexts. For this, we conducted two experiments, where each experiment tested one specific hypothesis. In the first experiment (H1), we assessed how different experiential beer packaging sounds would shape consumers’ general emotions and sensory expectations when presented across different frequency and sound pressure ranges. In the second experiment (H2), we analyzed how the sounds that prompted the most favorable results in the first experiment, when presented with the image of a NAB, would influence emotions and sensory expectations related to such NAB in a digital environment.

**Hypothesis** **1** **(H1).** *Experiential beer packaging sounds will distinctively modulate emotional state (i.e., positive vs. negative), as well as sensory expectations (i.e., sweetness, bitterness, alcohol strength, refreshing)*.

**Hypothesis** **2** **(H2).** *The experiential beer packaging sound(s) that evoked more positive scores in H1 will positively modulate the scores associated with a digital image of a NAB*.

## 2. Experiment 1

### 2.1. Materials and Methods

#### 2.1.1. Participants

One hundred and ninety-two (192) participants were directly invited to join Experiment 1 (gender-balanced sample). Responders were all over 18 years old (average age = 21.32 years; SD = 4.70) and were primarily undergraduate students from Universidad de Los Andes (Colombia). The sample size was calculated using a power analysis based on Friedman’s simplified determinations of statistical power [33]. Considering a 95% confidence level (α = 0.05), an effect size of 0.2, and power of 80%, the estimated sample size needed was set at approximately 191 individuals.

#### 2.1.2. Materials and Stimuli

**Figure 1:**
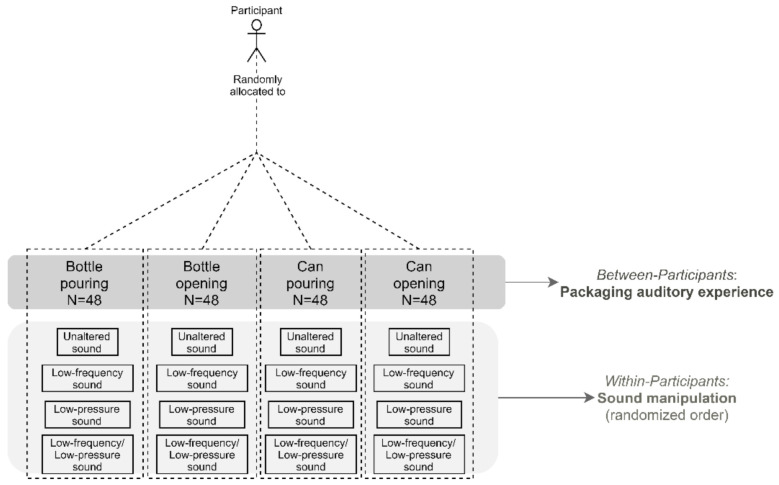
Design of Experiment 1, including the between- (packaging auditory experience) and within (sound manipulation)-participants conditions.

Pouring and opening sounds of a beer can and bottle were used as stimuli in Experiment 1. Two previous studies were used as support for the choice of such experiential beer packaging sounds. On the one hand, Spence et al. [16] suggested that the typical beverage product experiential sounds conveying important information are opening and pouring sounds. On the other hand, according to Almiron et al. [17], opening sounds may play a unique role in the beer experience since they are the first noticeable sounds, setting product-related expectations that may anchor the subsequent tasting experience. In fact, the sounds used in Experiment 1 were taken from Almiron et al.’s study [17]. Each of these experiential beer packaging sounds (opening and pouring) had four different versions, concerning sound pressure level (minus 15 dB for the softer sounds) and frequency ranges (minus six semitones, for the lower frequency sounds; see Figure 1). Hence, in total, sixteen auditory stimuli were used in Experiment 1. The stimuli can be accessed at https://osf.io/ve3ap/ (accessed on 19 August 2021).

#### 2.1.3. Experimental Design

As shown in Figure 1, Experiment 1 was based on a mixed-model experimental design. Here, four between-participants conditions were implemented. These conditions included a sound representing a typical beer packaging auditory experience (bottle pouring, bottle opening, can pouring, and can opening). For each between-participants condition, each participant was exposed to four versions of the same sound manipulated in terms of frequency and sound pressure (unaltered, low-frequency, low-pressure, and low-frequency/low-pressure sound).

#### 2.1.4. Procedure

Experiment 1′s survey was administrated on Qualtrics (https://www.qualtrics.com, accessed on 19 August 2021), and designed to last for approximately 10 min. Participants that agreed to join the study, providing their informed consent, were instructed to use headphones at all times and were randomly allocated to one of the four existing between-participants conditions. Each participant had to pass through an audio calibration process prior to the beginning of the main experiment. This calibration involved listening to, and locating, the balance of a stereo sound of a bird chipping, while setting the hearing volume at a comfortable level. This calibration process also worked as a validation step to double-check if participants were using the headphones while answering the questionnaire.

During the main experiment, the participants listened to each of the four within-participants experimental sounds presented in random order. Participants were told they would hear a sound of a beer being opened (or poured) and were instructed to play the sound. After listening to each sound as often as they wanted, they were asked to answer how they felt after listening to the sound, rating 16 emotional terms presented in random order. Afterward, the participants also had to rate to what extent each sound was associated with four sensory parameters related to a beer experience (see Measures Section (Section 2.1.5) below for a more detailed explanation of the dependent variables).

Finally, the participants were asked about their demographic profile while also passing through additional validation steps to double-check if they were paying full attention during the experiment. This study was approved by the Universidad de los Andes ethics committee under Act 1357 of 2021.

#### 2.1.5. Measures

In this study, we assessed two main dimensions of dependent variables: emotional and sensory ones. Concerning emotional scores, 16 terms were adapted from Silva et al. [11], which reported a list of emotions consistently associated with beer experiences. These 16 emotions were further subdivided into eleven positive (amused, calmed, comforted, curious, energetic, excited, friendly, good, joyful, pleased, responsible), one neutral (rational), and four negative (disappointed, grumpy, restless, sad) ones. Each emotion was evaluated by the participants based on the following instruction: “After hearing this sound, think about how it made you feel, and complete the following sentence: ‘I FEEL _____.’ (e.g., I FEEL AMUSED)”. Concerning the sensory scores, participants were asked to indicate how refreshing, sweet, bitter, and strong (as in beer alcohol percentage) they expected the beer associated with the sound they were listening to be. All answers were based on a 7-point scale, with 1 being “not at all” and 7 “very much”.

#### 2.1.6. Data Analysis

All data analyses were performed using IBM SPSS 26.0. Main effects were tested using the repeated-measures general linear model with sound manipulation (unaltered, low-frequency, low-pressure, and low-frequency and pressure) as a within-participants factor, and the packaging auditory experience (bottle pouring, bottle opening, can pouring, and can opening) as a between-participants factor. As for the dependent variables, two independent models were executed to observe the emotion scores, and the sensory expectations, separately. Pairwise comparisons were Bonferroni-corrected. Since gender was balanced and age variance was relatively low, they were not included as covariates during data analyses.

### 2.2. Results

#### 2.2.1. Emotion Scores

The results of the multivariate tests (Pillai’s Trace) revealed that there was a main effect of beer packaging auditory experience at the between-participants level (F(48, 525) = 2.05; *p* < 0.01; η^2^p = 0.16), and of manipulation at the within-participants level (F(48, 141) = 2.67; *p* < 0.01; η^2^p = 0.48). No interaction effects were found for packaging auditory experience and sound manipulation (F(144, 429) = 0.90; *p* = 0.763; η^2^p = 0.23).

Table 1 shows the univariate tests at the within-participants level (Greenhouse–Geisser-corrected). There was a main effect of sound manipulation on specific positive (amused (*p* < 0.01), comforted (*p* < 0.01), curious (*p* < 0.01), energetic (*p* < 0.01), excited (*p* < 0.01), friendly (*p* < 0.01), good (*p* < 0.01), joyful (*p* < 0.01), pleased (*p* < 0.01), and responsible (*p* < 0.05)) and negative (disappointed (*p* < 0.01), grumpy (*p* < 0.05)) emotion scores. No effects of sound manipulation were found for calmed, restless, and rational scores.

Table 2 shows the univariate tests at the between-participants level. There was a main effect of packaging auditory experience on specific positive (amused (*p* < 0.05), calmed (*p* < 0.01), comforted (*p* < 0.01), curious (*p* < 0.05), excited (*p* < 0.01), friendly (*p* < 0.01), good (*p* < 0.01), joyful (*p* < 0.05), and pleased (*p* < 0.01)), neutral (rational (*p* < 0.05)), as well as negative (disappointed (*p* < 0.01), grumpy (*p* < 0.01), restless (*p* < 0.01), and sad (*p* < 0.05)) emotion scores. No effects of packaging auditory experience were found for energetic and responsible scores.

The overall evidence obtained through these pairwise comparisons points to the bottle pouring sound as the one evoking the highest scores across the positive emotions dimension, followed by the can pouring and bottle opening sounds. Note that the sound of a can opening elicited the lowest scores on most positive emotion scores (see Table A1 in Appendix A). When it comes to the sound manipulation condition, the unaltered sound generally elicited the highest scores on the positive emotions, followed by its low-frequency version. Low-frequency/low-pressure and low-pressure sound versions evoked the lowest scores on positive emotion scores (see Table A2 in Appendix A). Concerning the negative emotions dimension, the bottle pouring sound elicited the lowest scores, followed by the bottle opening sound. The sounds of can opening and can pouring evoked the overall highest scores on the negative emotions (see Table A1 in Appendix A). When it comes to the sound manipulation condition, the unaltered and low-pressure sound versions elicited the lowest scores on negative emotions, followed by the low-frequency version of the sound. The low-frequency/low-pressure sound version evoked the highest scores on negative emotions and the responsible emotion score (see Table A2 in Appendix A). Finally, it is noteworthy that rational scores were the only ones whose evidence was not conclusive.

#### 2.2.2. Sensory Expectations

The results of the multivariate tests (Pillai’s Trace) revealed that there was a main effect of packaging auditory experience at the between-participants level (F(12, 561) = 3.44; *p* < 0.01; η^2^p = 0.07), and of sound manipulation at the within-participants level (F(12, 177) = 6.42; *p* < 0.01; η^2^p = 0.30). No interaction effects were found for packaging auditory experience and sound manipulation (F(36,537) = 0.98; *p* = 0.500; η^2^p = 0.06).

Table 3 shows the results of the univariate tests at the within-participants level (Greenhouse–Geisser-corrected). Here, the main effect of sound manipulation on every sensory expectation was detected (alcohol level (*p* < 0.01), refreshing (*p* < 0.01), sweetness (*p* < 0.01), and bitterness (*p* < 0.01)).

Table 4 shows the results of the univariate tests at the between-participants level, where the main effect of packaging auditory experience sounds was found only for the refreshing scores (*p* < 0.01). No effects of packaging auditory experiences were found for the alcohol level, sweetness, and bitterness sensory scores.

The corresponding pairwise comparisons showed that the bottle pouring sound evoked the highest refreshing scores compared to other packaging sounds (see Table A3 and Table A4 in Appendix A). Concerning the sound manipulation condition, the unaltered and low-frequency versions of the sounds boosted more refreshing and alcohol strength scores when compared to the low-pressure and low-frequency/low-pressure versions. The sweetness scores were higher with the low-pressure version of the sound than with the low-frequency one. Conversely, the bitterness scores were higher with the low-frequency version of the sound than the low-pressure one. The unaltered and the low-frequency/low-pressure versions did not show significant differences concerning sweetness and bitterness scores.

## 3. Experiment 2

### 3.1. Participants

Four hundred participants joined Experiment 2 (female = 38%). Participants were all over 18 years old (mean age: 25.4 years; SD = 8.4), and 61% reported drinking beer at least once a month, while 80% reported rarely drinking NAB (i.e., less than once a month). All participants were recruited via Prolific, which is a British database company specialized in setting panels for online experiments (https://www.prolific.co/, accessed on 19 August 2021). Each participant was remunerated with approximately US$0.95 for their participation in this study. Considering a 95% confidence level (α = 0.05), an effect size of 0.15, and power of 80%, the estimated sample size needed was set at approximately 343 individuals [33].

### 3.2. Materials and Stimuli

#### 3.2.1. Auditory Stimuli

**Figure 2:**
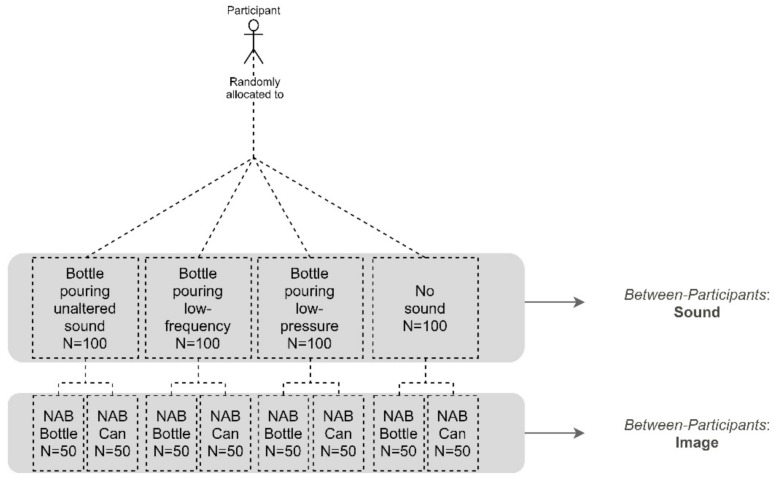
Design of Experiment 2 including the two types of stimuli (sound and image) employed simultaneously in the between-participants condition.

The beer bottle pouring sound showed general evidence of triggering the most positive effects in Experiment 1 (see results of Experiment 1). First, the unaltered version of the beer bottle pouring sound triggered the highest scores on most positive emotions. Secondly, the low-frequency version of this beer bottle pouring sound prompted the highest scores on alcohol strength and bitterness expectations. Third, the low-pressure version of the same beer bottle pouring sound triggered the highest scores on sweetness expectation. Hence, these three sounds were chosen as auditory stimuli for Experiment 2 (see Figure 2).

#### 3.2.2. Visual Stimuli

**Figure 3:**
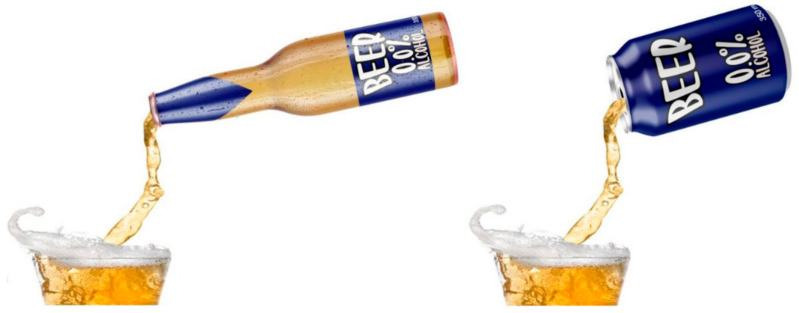
Visual stimuli used in Experiment 2. Left: NAB bottle pouring. Right: NAB can pouring.

Each of the chosen three experimental sounds were presented with a customized NAB brand-free image, representing a digital NAB experience of beer poured into glassware (see Figure 3 for both bottle and can versions of NAB used in Experiment 2). The labels had a blue background since NAB versions of most well-known beer brands usually include this color as part of their labeling. Importantly, blue background labels usually represent lighter and healthier product versions [34,35,36]. This experimental label also contained information that the poured product was a 0.0% alcohol type of beer (NAB). Both images were produced using Adobe Photoshop CS6 software and relied on baseline images under creative commons licensing.

### 3.3. Methods

#### 3.3.1. Design

In Experiment 2, each participant was randomly exposed to one out of the three auditory conditions selected from Experiment 1, plus a control no-sound condition (unaltered version of a beer bottle pouring, low-frequency version of beer bottle pouring, low-pressure version of a beer bottle pouring, or no sound at all). To simulate a NAB online shopping experience, each sound was presented along with one of the existing NAB images, depending on which condition the participant was randomly assigned to (see Figure 2 for an overview of this experimental design). In total, there were eight experimental between-participants conditions (combining the existing four auditory cues, and the two available images).

#### 3.3.2. Procedure

Experiment 2 was delivered via Qualtrics (https://www.qualtrics.com, accessed on 19 August 2021), and designed to last for approximately 5 min. The procedure of Experiment 2 was the same as in Experiment 1, with the main difference being that each participant had only one stimulus to evaluate (hence, no within-participants comparisons), and such stimulus was audiovisual (meaning image plus sound). Moreover, at the end of the survey of Experiment 2, the participants were asked about their beer and NAB consumption habits.

#### 3.3.3. Data Analysis

Statistical analyses were performed using IBM SPSS 26.0. Main effects and interactions were assessed using two independent multivariate ANOVA general linear models. Each independent ANOVA calculated the emotion scores and sensory expectations, independently, with two fixed factors (image and sound). Age, gender, and beer/NAB consumption habits were included as covariates. Pairwise comparisons were Bonferroni-corrected. To assess how the covariates affected the emotion scores, correlations were obtained via Pearson’s method.

### 3.4. Results

#### 3.4.1. Emotion Scores

Table 5 shows the results of the multivariate tests (Pillai’s Trace). Here, a main effect of sound (*p* < 0.01) was detected. A main effect of NAB consumption habit (*p* < 0.05) and age (*p* < 0.01) on results was also detected. No main effect was found for the image (*p* = 0.073) or its interaction with sound (*p* = 0.580). There was no effect of gender (*p* = 0.065) and beer consumption habit (*p* = 0.114) on results.

The univariate tests revealed a main effect of sound on specific positive (calmed (*p* < 0.01), comforted (*p* < 0.01), excited (*p* < 0.01), friendly (*p* < 0.05), good (*p* < 0.01), joyful (*p* < 0.01), pleased (*p* < 0.01), and responsible (*p* < 0.01)) and negative (sad (*p* < 0.01), grumpy (*p* < 0.01), and disappointed (*p* < 0.01)) emotion scores. These results also showed a main effect of the NAB consumption habit covariate on specific positive (comforted (*p* < 0.05), energetic (*p* < 0.05), excited (*p* < 0.01), good (*p* < 0.05), responsible (*p* < 0.05)) and neutral (rational (*p* < 0.01)) emotion scores (see Table 6). Moreover, the age covariate showed a main effect on comforted (*p* < 0.05), energetic (*p* < 0.05), and grumpy (*p* < 0.01) emotion scores. Amused, curious, and restless emotion scores did not prompt effects.

**Figure 4:**
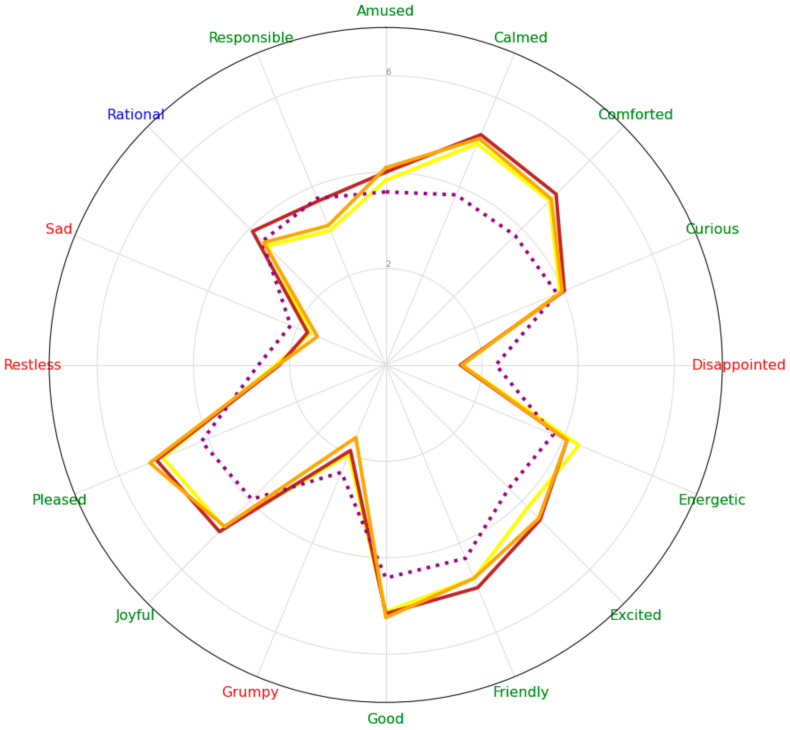
Radar chart with the mean averages of auditory conditions for every emotion score in Experiment 2. The purple/dotted-line represents the no-sound condition. The brown line, the low-pressure one. The yellow line, the low-frequency sound condition. The orange line, the unaltered sound condition.

Figure 4 depicts a visual representation of the participants’ scores in Experiment 2, concerning the emotion scores, and under each sound condition. Here, it is possible to visually appreciate how the emotion scores of the participants under the no-sound condition stand in sharp contrast to the participants who listened to a sound while rating.

The corresponding pairwise comparisons showed overall evidence that most positive emotion scores were higher under any bottle pouring sound conditions when compared to the no-sound condition. The no-sound condition, in turn, elicited the highest scores concerning negative emotions, followed by low-frequency and low-pressure sound versions. The unaltered version of the sound elicited the lowest scores on the negative emotions. Responsible emotion scores were also higher under the no-sound condition (see Table A5 in Appendix A). Pearson correlation analysis was conducted for age and NAB consumption habit covariates on the emotion scores that showed significant main effects (Table 6). The results of these correlations suggest that the more NAB consumption frequency, the higher scores on positive and neutral emotions (amused (r = 0.11), comforted (r = 0.17), energetic (r = 0.14), excited (r = 0.23), good (r = 0.17), rational (r = 0.19), and responsible (r = 0.11)). There were no conclusive results concerning the effect of age on emotion scores.

#### 3.4.2. Sensory Expectations

Table 7 shows the results of the multivariate tests for the sensory expectation scores (Pillai’s Trace). Here, there was a main effect of sound (*p* < 0.01) and NAB consumption habit (*p* < 0.01) on results. No effects were found for the image (*p* = 0.654) or its interaction with sound (*p* = 0.594). There were also no effects of gender (*p* = 0.108), age (*p* = 0.239), or beer consumption habit (*p* = 0.402) on results.

Table 8 shows the univariate test results. Here, there was a main effect of sound, and specifically on the refreshing expectations scores (*p* < 0.01). NAB consumption habit was found to affect the sweetness expectations scores (*p* < 0.01). No effects were found for the bitterness expectations.

The unaltered bottle pouring sound and its low-pressure version elicited the highest refreshing expectations, followed by its low-frequency version. The no-sound condition evoked the lowest refreshing scores (see Table A6 in Appendix A). Pearson correlation analysis was conducted to assess the effect of NAB consumption habit on sweetness expectation scores, which prompted a significant main effect in the univariate tests (see Table 8). These correlations suggest that the more NAB consumption frequency, the higher scores on sweetness expectation (r = 0.20).

## 4. Summary of All the Obtained Results

The results obtained in Experiment 1 allowed us to select three particular experiential beer sounds that most positively influenced participants’ emotions and sensory expectations (unaltered bottle pouring, low-frequency bottle pouring, and low-pressure bottle pouring sounds). In Experiment 2, we used those three selected sounds and assessed their role in a NAB simulated digital shopping experience. Principally, Experiment 2 results revealed that the unaltered bottle pouring sound triggered the most positive and the least negative emotions, as well as the highest refreshing expectations for the image of a NAB, compared to the other three auditory conditions (and regardless of whether the NAB was a can or a bottle). Moreover, in Experiment 2, regardless of sound or image condition, the participants that reported more often consuming NAB generally felt more positive and neutral (i.e., rational) in terms of emotions, while also generally expecting a sweeter NAB.

## 5. Discussion

This study generally focused on assessing how specific sounds associated with a beer packaging experience may influence consumer emotions and sensory expectations on a simulated digital shopping experience of a NAB. We were inspired by the possibility of using auditory cues to enable brands and organizations to more effectively nudge consumers to consider NAB as part of their evoked set during decision-making tasks related to beers and/or overall drink choices. Customizing packaging auditory experiences may be crucial due to the proven existing link between product sounds and emotions/expectations related to product consumption [37].

Overall, the results showed that particular emotions and sensory expectations were modulated depending on the auditory condition to which the participants were exposed. More specifically, by presenting an unaltered bottle pouring sound along with the image of a NAB being poured in a glass, we were able to counteract some of the negative emotions that are commonly associated with the experience of a NAB, while at the same time enhancing most of the positive emotion scores being assessed throughout the study. Note that the same bottle pouring sound significantly enhanced refreshing expectations for the NAB as well.

### 5.1. The Positive Effect That Experiential Beer Sounds Can Bring to the Experience of Consumers (H1)

Experiment 1 was useful to identify which beer packaging sounds, customized in terms of acoustic parameters, might prompt significant and specific emotional effects and sensory expectations in the experience of consumers. We, therefore, showed that different experiential beer packaging sounds could modulate emotional state and sensory expectations in very particular ways (H1). The obtained results were somehow in line with previous research, where, for instance, the same unaltered bottle pouring sound used in this study was previously framed as more premium than other experiential beer packaging sounds [17]. In this previous study, the authors also suggested that the most premium sounds tend to be more semantically associated with positive adjectives, such as alive, good, nice, and happy. Hence, our results add value to the existing literature by providing insights on how a bottle pouring sound can enhance positive emotions and sensory expectations on beer product experiences. Such findings may be helpful while promoting specificities of the value proposition of any beer, including NAB.

Previous research has also shown that when consumers are induced into negative emotions (e.g., via music or movie clips), they tend to perceive a beer’s flavor as more alcoholic, less sweet, and/or more bitter [38,39]. In Experiment 1, the auditory conditions that participants reported as the most negative were the low-frequency/low-pressure can-opening/can-pouring ones. However, in such conditions, the alcohol content expectation scored by the participants was the lowest, and there were no other main effects observed in such auditory conditions on bitterness or sweetness expectations.

In another similar study, the freshness of carbonated beverages was more associated with high-pitched pouring sounds and small bubbles when compared to low-pitched pouring sounds and big bubbles [31]. However, in the sound manipulation condition of Experiment 1, the refreshing expectation was enhanced with the unaltered (vs. low-pressure) sound. Moreover, in Experiment 2, the refreshing expectations were significantly enhanced when participants listened to the unaltered (vs. low-frequency) sound, which is in line with Roque et al.’s study [31].

Hence, in the present study, we could conclude that the priming effect of a NAB image, as part of Experiment 2, improved the overall conception of this digital beer experience, while more accordingly shaping participants’ emotional and sensory expectations than in Experiment 1.

### 5.2. Using Experiential Beer Sounds to Nudge Consumers towards NAB Choices in Digital Shopping Environments (H2)

In Experiment 2, we empirically showed that the usage of beer bottle pouring sounds could enhance positive emotions and refreshing expectations towards NAB in digital shopping environments. Here, we showed that the sound that evoked more positive emotions in Experiment 1 positively modulated the listener’s emotions towards a NAB as well (H2). To the best of our knowledge, this is the first experiment where product sounds are being used to evoke positive impressions on NAB. Hence, we believe that these results may be important to those interested in the existing literature that focuses on the role of background sound and music on consumers’ decision-making tasks associated with different dietary foods or beverages [40,41].

Notably, the unaltered beer bottle pouring sound enhanced the refreshing expectations of NAB, and such effects were obtained regardless of the NAB image shown to participants (i.e., beer or can). On the one hand, as explained before, the obtained results are in line with previous research, where it has been shown that high-pitched sounds representing small bubbling (vs. low-pitched sounds/big bubbling) tend to be more congruent with fresher sparkling beverages [31]. On the other hand, this finding may also indicate that consumers can disentangle the image from the sound while shopping in e-commerce, thus not framing the multisensory experience of the image of a can, when accompanied by a beer bottle pouring sound, as semantically incongruent. In other words, in this digital NAB experience, the bottle pouring sound may have acted as an standalone soundscape rather than as a part of the audiovisual experience associated with the product’s image (see [27,42]).

On top of that, in Experiment 2, the participants that reported often consuming NAB generally acknowledged feeling more rational and responsible. Here, such participants also rated a NAB as generally sweeter. These findings seem logical since rational and responsible emotions are commonly associated with NAB consumption [10], and NAB is often considered sweeter than its alcoholic counterpart [43].

### 5.3. General Implications

It is well-known that emotions play an important role in purchase decision-making tasks [44,45]. As a matter of fact, in previous research, Silva et al. [11] found that a NAB was more liked when it was consumed “disguised” as an alcoholic beer, where participants felt more fulfilled under such a situation. The label of NAB, on its own, prompts negative (sad, disappointed, and grumpy) and weakens positive (comforted, exuberant, good, happy, joyful, and loving) emotions. In addition, consuming NAB evokes neutral and negative emotional responses, such as rational, conscious, and disappointed [10]. In this way, our results show that a customized experiential beer packaging sound can be employed to counteract such negativity commonly associated with NAB experiences.

Based on the obtained results, brands interested in more positively promoting NAB in a virtual shopping environment may feel encouraged to involve product packaging sounds as part of the customer’s digital shopping experience. In particular, here, it has been shown that a beer bottle pouring sound can be used to elicit positive emotions during a NAB online shopping experience. In addition, a bottle pouring sound cannot only diminish negative emotions, but it may also be useful to boost NAB refreshing expectations. Thus, the present study broadens the scope of the current state-of-the-art by showing the effect that product sounds can have on emotions and sensory expectation variables in online retailing associated with NAB. Such variables could act as a support for crucial consumption ones as well, such as willingness to pay (see [20]).

As a matter of fact, healthy foods/beverage producers may consider promoting nutritional values (i.e., lower alcohol/calories/fat/sugar/salt content) via packaging sounds. When it comes to promoting healthy sparkling beverages, such as NAB, it has been suggested that people automatically tend to associate higher pitch and quieter sounds with a lower-calorie or reduced sugar product—although, as far as we are aware, the latter has not been empirically proven yet [15].

In brief, this study can motivate beer brands and organizations to consider including sound during packaging design strategies to enhance the multisensory shopping experience of their healthier product categories, such as NAB.

### 5.4. Limitations and Future Work

In online retail environments, products are not assessed individually but are usually compared across competing products within a specific category. Since our study only involved NAB, future similar work could examine the effects of similar sounds of a beer packaging experience when different types of beers are presented simultaneously to the participants. In this way, the setting would be more realistic, and emotions could play a different role. For example, future research could compare NAB paired with a pouring sound vs. regular beer with no sound to support the idea that sounds could not only increase the positive emotions related to NAB but could also encourage the preference of NAB over alcoholic beer. Additionally, variables such as liking, familiarity with the product, and willingness to pay may also be critical to measure along with emotions and sensory expectations in similar future assessments (see [20]).

Concerning methodology, we only manipulated the original experiential sounds towards lower frequency and sound pressure ranges. Future similar work could do the opposite by focusing on the higher frequency/pressure components of the sound stimuli. For instance, previous research found semantic associations between higher-pitched can opening sounds and specific comfort scores [18]. It may also be interesting to mix those experiential beer sounds with other sound cues that may be part of more realistic virtual retail experiences, such as background noise and/or music.

## Figures and Tables

**Table 1:** Summary of the results of univariate tests (Greenhouse–Geisser-corrected) of Experiment 1 at the within-participants level (sound manipulation condition) on each emotion score.

Source		Degrees of Freedom	Mean Square	F	*p*-Value	Partial Eta Squared
Sound manipulation	Amused	2.71	34.89	20.11	<0.01	0.10
	Calmed	2.97	2.43	1.41	0.24	0.01
	Comforted	2.87	16.40	8.65	<0.01	0.04
	Curious	2.84	12.12	6.61	<0.01	0.03
	Disappointed	2.79	16.40	8.69	<0.01	0.04
	Energetic	2.76	43.24	25.66	<0.01	0.12
	Excited	2.68	22.09	11.56	<0.01	0.06
	Friendly	2.70	17.08	11.23	<0.01	0.06
	Good	2.76	8.84	5.83	<0.01	0.03
	Grumpy	2.89	3.97	2.97	0.03	0.02
	Joyful	2.73	21.78	13.34	<0.01	0.07
	Pleased	2.82	25.54	12.55	<0.01	0.06
	Restless	2.95	0.60	0.38	0.76	<0.01
	Sad	2.88	9.41	9.51	<0.01	0.05
	Rational	2.95	1.24	1.03	0.38	0.01
	Responsible	2.92	3.11	3.00	0.03	0.02

**Table 2:** Summary of the results of the univariate tests of Experiment 1 at the between-participants level (packaging auditory experience condition) on each emotion score.

Source		Degrees of Freedom	Mean Square	F	*p*-Value	Partial Eta Squared
Packaging auditory experience	Amused	3.00	34.77	3.81	0.01	0.06
	Calmed	3.00	31.70	4.24	0.01	0.06
	Comforted	3.00	47.63	6.06	<0.01	0.09
	Curious	3.00	25.01	2.74	0.04	0.04
	Disappointed	3.00	35.72	5.97	<0.01	0.09
	Energetic	3.00	17.38	1.94	0.12	0.03
	Excited	3.00	46.46	5.08	<0.01	0.07
	Friendly	3.00	42.40	4.44	<0.01	0.07
	Good	3.00	49.98	7.13	<0.01	0.10
	Grumpy	3.00	20.52	4.31	0.01	0.06
	Joyful	3.00	29.40	3.29	0.02	0.05
	Pleased	3.00	62.96	7.93	<0.01	0.11
	Restless	3.00	27.44	4.54	<0.01	0.07
	Sad	3.00	10.55	2.82	0.04	0.04
	Rational	3.00	23.38	3.15	0.03	0.05
	Responsible	3.00	10.23	1.31	0.27	0.02

**Table 3:** Summary of the results of the univariate tests (Greenhouse–Geisser-corrected) of Experiment 1 at the within-participants level (sound manipulation condition) on each sensory score.

Source		Degrees of Freedom	Mean Square	F	*p*-Value	Partial Eta Squared
Sound manipulation	Alcohol	2.64	17.60	12.04	<0.01	0.06
	Refreshing	2.60	57.69	23.97	<0.01	0.11
	Sweetness	2.94	6.96	3.98	0.01	0.02
	Bitterness	2.98	7.89	3.88	0.01	0.02

**Table 4:** Summary of the results of the univariate tests of Experiment 1 at the between-participants level (packaging auditory experience condition) on each sensory score.

Source		Degrees of Freedom	Mean Square	F	*p*-Value	Partial Eta Squared
Packaging auditory experience	Alcohol	3.00	9.14	0.87	0.46	0.01
	Refreshing	3.00	70.78	9.64	<0.01	0.13
	Sweetness	3.00	15.65	2.29	0.08	0.04
	Bitterness	3.00	13.91	1.58	0.20	0.02

**Table 5:** Summary of the results of the Pillai’s Trace multivariate tests for the emotion scores in Experiment 2.

Effect	Value	F	Hypothesis Degrees of Freedom	Error Degrees of Freedom	*p*-Value	Partial Eta Squared
Beer consumption habit	0.06	1.61	16.00	373.00	0.06	0.06
NAB consumption habit	0.07	1.82	16.00	373.00	0.03	0.07
Age	0.12	3.16	16.00	373.00	<0.01	0.12
Gender	0.06	1.45	16.00	373.00	0.11	0.06
Image	0.06	1.57	16.00	373.00	0.07	0.06
Sound	0.28	2.43	48.00	1125.00	<0.01	0.09
Image * Sound ^1^	0.12	0.95	48.00	1125.00	0.58	0.04

*^,1^ Interaction effect between image and sound factors.

**Table 6:** Summary of the results of the univariate tests at the between-participants level on each emotion score in Experiment 2.

Source		Degrees of Freedom	Mean Square	F	*p*-Value	Partial Eta Squared
NAB consumption habit	Amused	1.00	9.12	3.10	0.08	0.01
	Calmed	1.00	3.42	1.32	0.25	0.00
	Comforted	1.00	16.19	6.53	0.01	0.02
	Curious	1.00	0.60	0.21	0.65	0.00
	Disappointed	1.00	0.57	0.35	0.55	0.00
	Energetic	1.00	9.37	3.96	0.05	0.01
	Excited	1.00	32.32	12.18	<0.01	0.03
	Friendly	1.00	0.14	0.06	0.80	0.00
	Good	1.00	11.19	5.70	0.02	0.01
	Grumpy	1.00	0.54	0.31	0.58	0.00
	Joyful	1.00	5.09	2.11	0.15	0.01
	Pleased	1.00	6.46	3.06	0.08	0.01
	Restless	1.00	0.07	0.03	0.86	0.00
	Sad	1.00	0.09	0.06	0.81	0.00
	Rational	1.00	26.11	11.04	<0.01	0.03
	Responsible	1.00	12.94	4.72	0.03	0.01
Age	Amused	1.00	0.32	0.11	0.74	0.00
	Calmed	1.00	3.67	1.41	0.24	0.00
	Comforted	1.00	11.60	4.68	0.03	0.01
	Curious	1.00	8.60	2.96	0.09	0.01
	Disappointed	1.00	0.96	0.59	0.44	0.00
	Energetic	1.00	11.99	5.08	0.02	0.01
	Excited	1.00	0.11	0.04	0.84	0.00
	Friendly	1.00	0.36	0.16	0.69	0.00
	Good	1.00	0.52	0.26	0.61	0.00
	Grumpy	1.00	14.40	8.27	<0.01	0.02
	Joyful	1.00	5.07	2.10	0.15	0.01
	Pleased	1.00	0.14	0.07	0.80	0.00
	Restless	1.00	4.31	1.97	0.16	0.01
	Sad	1.00	1.02	0.66	0.42	0.00
	Rational	1.00	2.70	1.14	0.29	0.00
	Responsible	1.00	7.93	2.89	0.09	0.01
Sound	Amused	3.00	4.05	1.38	0.25	0.01
	Calmed	3.00	36.32	14.01	<0.01	0.10
	Comforted	3.00	25.63	10.34	<0.01	0.07
	Curious	3.00	0.34	0.12	0.95	0.00
	Disappointed	3.00	12.94	8.03	<0.01	0.06
	Energetic	3.00	3.85	1.63	0.18	0.01
	Excited	3.00	13.99	5.27	<0.01	0.04
	Friendly	3.00	6.52	2.91	0.03	0.02
	Good	3.00	11.71	5.97	<0.01	0.04
	Grumpy	3.00	11.07	6.36	<0.01	0.05
	Joyful	3.00	15.59	6.47	<0.01	0.05
	Pleased	3.00	24.03	11.39	<0.01	0.08
	Restless	3.00	4.15	1.89	0.13	0.01
	Sad	3.00	6.44	4.20	0.01	0.03
	Rational	3.00	2.93	1.24	0.30	0.01
	Responsible	3.00	14.56	5.31	<0.01	0.04

**Table 7:** Summary of the results of the Pillai’s Trace multivariate tests for the sensory scores in Experiment 2.

Effect	Value	F	Hypothesis Degrees of Freedom	Error Degrees of Freedom	*p*-Value	Partial Eta Squared
Beer consumption habit	0.01	0.98	3.00	386.00	0.40	0.01
NAB consumption habit	0.05	6.82	3.00	386.00	<0.01	0.05
Age	0.01	1.41	3.00	386.00	0.24	0.01
Gender	0.02	2.04	3.00	386.00	0.11	0.02
Image	0.00	0.54	3.00	386.00	0.65	0.00
Sound	0.06	2.68	9.00	1164.00	<0.01	0.02
Image * Sound ^1^	0.02	0.82	9.00	1164.00	0.59	0.01

*^,1^ Interaction effect between image and sound factors.

**Table 8:** Summary of the results of the univariate tests at the between-participants level on each sensory expectation in Experiment 2.

Source		Degrees of Freedom	Mean Square	F	*p*-Value	Partial Eta Squared
NAB consumption habit	Refreshing	1.00	2.02	1.33	0.25	0.00
	Sweetness	1.00	41.41	15.84	<0.01	0.04
	Bitterness	1.00	6.33	2.13	0.15	0.01
Sound	Refreshing	3.00	9.28	6.10	<0.01	0.05
	Sweetness	3.00	2.26	0.87	0.46	0.01
	Bitterness	3.00	5.52	1.86	0.14	0.01

## Data Availability

The original contributions presented in the study are included in the article. Further inquiries can be directed to the corresponding author.

## Appendix A

**Table A1:** Summary of the results of the pairwise comparisons of Experiment 1 within-participants condition (sound manipulation) for emotion scores.

Measure	Stimuli		Mean Difference	Std. Error	*p*-Value
Amused	Low-frequency	Low-frequency/low-pressure	0.73	0.14	<0.01
		Low-pressure	0.46	0.14	0.01
		Unaltered	−0.14	0.11	1.00
	Low-frequency/low-pressure	Low-frequency	−0.73	0.14	<0.01
		Low-pressure	−0.27	0.11	0.10
		Unaltered	−0.87	0.13	<0.01
	Low-pressure	Low-frequency	−0.46	0.14	0.01
		Low-frequency/low-pressure	0.27	0.11	0.10
		Unaltered	−0.60	0.13	<0.01
	Unaltered	Low-frequency	0.14	0.11	1.00
		Low-frequency/low-pressure	0.87	0.13	<0.01
		Low-pressure	0.60	0.13	<0.01
Calmed	Low-frequency	Low-frequency/low-pressure	0.03	0.13	1.00
		Low-pressure	−0.18	0.13	1.00
		Unaltered	−0.18	0.13	1.00
	Low-frequency/low-pressure	Low-frequency	−0.03	0.13	1.00
		Low-pressure	−0.21	0.13	0.68
		Unaltered	−0.21	0.13	0.69
	Low-pressure	Low-frequency	0.18	0.13	1.00
		Low-frequency/low-pressure	0.21	0.13	0.68
		Unaltered	0.00	0.14	1.00
	Unaltered	Low-frequency	0.18	0.13	1.00
		Low-frequency/low-pressure	0.21	0.13	0.69
		Low-pressure	0.00	0.14	1.00
Comforted	Low-frequency	Low-frequency/low-pressure	0.49	0.14	<0.01
		Low-pressure	0.14	0.15	1.00
		Unaltered	−0.19	0.14	1.00
	Low-frequency/low-pressure	Low-frequency	−0.49	0.14	<0.01
		Low-pressure	−0.35	0.12	0.02
		Unaltered	−0.68	0.14	<0.01
	Low-pressure	Low-frequency	−0.14	0.15	1.00
		Low-frequency/low-pressure	0.35	0.12	0.02
		Unaltered	−0.32	0.14	0.14
	Unaltered	Low-frequency	0.19	0.14	1.00
		Low-frequency/low-pressure	0.68	0.14	<0.01
		Low-pressure	0.32	0.14	0.14
Curious	Low-frequency	Low-frequency/low-pressure	0.41	0.14	0.02
		Low-pressure	0.22	0.13	0.59
		Unaltered	−0.15	0.14	1.00
	Low-frequency/low-pressure	Low-frequency	−0.41	0.14	0.02
		Low-pressure	−0.19	0.12	0.73
		Unaltered	−0.56	0.15	<0.01
	Low-pressure	Low-frequency	−0.22	0.13	0.59
		Low-frequency/low-pressure	0.19	0.12	0.73
		Unaltered	−0.37	0.13	0.02
	Unaltered	Low-frequency	0.15	0.14	1.00
		Low-frequency/low-pressure	0.56	0.15	<0.01
		Low-pressure	0.37	0.13	0.02
Disappointed	Low-frequency	Low-frequency/low-pressure	−0.40	0.14	0.03
		Low-pressure	−0.05	0.14	1.00
		Unaltered	0.29	0.12	0.12
	Low-frequency/low-pressure	Low-frequency	0.40	0.14	0.03
		Low-pressure	0.35	0.12	0.03
		Unaltered	0.69	0.15	<0.01
	Low-pressure	Low-frequency	0.05	0.14	1.00
		Low-frequency/low-pressure	−0.35	0.12	0.03
		Unaltered	0.34	0.13	0.08
	Unaltered	Low-frequency	−0.29	0.12	0.12
		Low-frequency/low-pressure	−0.69	0.15	<0.01
		Low-pressure	−0.34	0.13	0.08
Energetic	Low-frequency	Low-frequency/low-pressure	0.70	0.13	<0.01
		Low-pressure	0.38	0.13	0.03
		Unaltered	−0.35	0.12	0.03
	Low-frequency/low-pressure	Low-frequency	−0.70	0.13	<0.01
		Low-pressure	−0.32	0.10	0.02
		Unaltered	−1.05	0.14	<0.01
	Low-pressure	Low-frequency	−0.38	0.13	0.03
		Low-frequency/low-pressure	0.32	0.10	0.02
		Unaltered	−0.73	0.13	<0.01
	Unaltered	Low-frequency	0.35	0.12	0.03
		Low-frequency/low-pressure	1.05	0.14	<0.01
		Low-pressure	0.73	0.13	<0.01
Excited	Low-frequency	Low-frequency/low-pressure	0.60	0.13	<0.01
		Low-pressure	0.30	0.14	0.17
		Unaltered	−0.11	0.12	1.00
	Low-frequency/low-pressure	Low-frequency	−0.60	0.13	<0.01
		Low-pressure	−0.30	0.11	0.05
		Unaltered	−0.71	0.15	<0.01
	Low-pressure	Low-frequency	−0.30	0.14	0.17
		Low-frequency/low-pressure	0.30	0.11	0.05
		Unaltered	−0.42	0.14	0.02
	Unaltered	Low-frequency	0.11	0.12	1.00
		Low-frequency/low-pressure	0.71	0.15	<0.01
		Low-pressure	0.42	0.14	0.02
Friendly	Low-frequency	Low-frequency/low-pressure	0.45	0.13	<0.01
		Low-pressure	0.06	0.12	1.00
		Unaltered	−0.23	0.11	0.21
	Low-frequency/low-pressure	Low-frequency	−0.45	0.13	<0.01
		Low-pressure	−0.39	0.10	<0.01
		Unaltered	−0.68	0.13	<0.01
	Low-pressure	Low-frequency	−0.06	0.12	1.00
		Low-frequency/low-pressure	0.39	0.10	<0.01
		Unaltered	−0.30	0.11	0.06
	Unaltered	Low-frequency	0.23	0.11	0.21
		Low-frequency/low-pressure	0.68	0.13	<0.01
		Low-pressure	0.30	0.11	0.06
Good	Low-frequency	Low-frequency/low-pressure	0.31	0.12	0.06
		Low-pressure	0.09	0.12	1.00
		Unaltered	−0.18	0.11	0.60
	Low-frequency/low-pressure	Low-frequency	−0.31	0.12	0.06
		Low-pressure	−0.22	0.11	0.24
		Unaltered	−0.50	0.13	<0.01
	Low-pressure	Low-frequency	−0.09	0.12	1.00
		Low-frequency/low-pressure	0.22	0.11	0.24
		Unaltered	−0.28	0.13	0.21
	Unaltered	Low-frequency	0.18	0.11	0.60
		Low-frequency/low-pressure	0.50	0.13	<0.01
		Low-pressure	0.28	0.13	0.21
Grumpy	Low-frequency	Low-frequency/low-pressure	−0.06	0.12	1.00
		Low-pressure	0.24	0.11	0.21
		Unaltered	0.18	0.12	0.78
	Low-frequency/low-pressure	Low-frequency	0.06	0.12	1.00
		Low-pressure	0.30	0.10	0.03
		Unaltered	0.23	0.12	0.32
	Low-pressure	Low-frequency	−0.24	0.11	0.21
		Low-frequency/low-pressure	−0.30	0.10	0.03
		Unaltered	−0.06	0.12	1.00
	Unaltered	Low-frequency	−0.18	0.12	0.78
		Low-frequency/low-pressure	−0.23	0.12	0.32
		Low-pressure	0.06	0.12	1.00
Joyful	Low-frequency	Low-frequency/low-pressure	0.58	0.13	<0.01
		Low-pressure	0.23	0.12	0.31
		Unaltered	−0.16	0.11	0.86
	Low-frequency/low-pressure	Low-frequency	−0.58	0.13	<0.01
		Low-pressure	−0.34	0.11	0.01
		Unaltered	−0.74	0.14	<0.01
	Low-pressure	Low-frequency	−0.23	0.12	0.31
		Low-frequency/low-pressure	0.34	0.11	0.01
		Unaltered	−0.40	0.13	0.02
	Unaltered	Low-frequency	0.16	0.11	0.86
		Low-frequency/low-pressure	0.74	0.14	<0.01
		Low-pressure	0.40	0.13	0.02
Pleased	Low-frequency	Low-frequency/low-pressure	0.62	0.14	<0.01
		Low-pressure	0.31	0.14	0.18
		Unaltered	−0.18	0.13	0.96
	Low-frequency/low-pressure	Low-frequency	−0.62	0.14	<0.01
		Low-pressure	−0.31	0.13	0.11
		Unaltered	−0.80	0.16	<0.01
	Low-pressure	Low-frequency	−0.31	0.14	0.18
		Low-frequency/low-pressure	0.31	0.13	0.11
		Unaltered	−0.50	0.14	<0.01
	Unaltered	Low-frequency	0.18	0.13	0.96
		Low-frequency/low-pressure	0.80	0.16	<0.01
		Low-pressure	0.50	0.14	<0.01
Restless	Low-frequency	Low-frequency/low-pressure	0.11	0.13	1.00
		Low-pressure	0.12	0.12	1.00
		Unaltered	0.10	0.12	1.00
	Low-frequency/low-pressure	Low-frequency	−0.11	0.13	1.00
		Low-pressure	0.01	0.13	1.00
		Unaltered	−0.01	0.13	1.00
	Low-pressure	Low-frequency	−0.12	0.12	1.00
		Low-frequency/low-pressure	−0.01	0.13	1.00
		Unaltered	−0.02	0.13	1.00
	Unaltered	Low-frequency	−0.10	0.12	1.00
		Low-frequency/low-pressure	0.01	0.13	1.00
		Low-pressure	0.02	0.13	1.00
Sad	Low-frequency	Low-frequency/low-pressure	−0.30	0.10	0.03
		Low-pressure	0.05	0.10	1.00
		Unaltered	0.22	0.09	0.11
	Low-frequency/low-pressure	Low-frequency	0.30	0.10	0.03
		Low-pressure	0.35	0.11	0.01
		Unaltered	0.52	0.10	<0.01
	Low-pressure	Low-frequency	−0.05	0.10	1.00
		Low-frequency/low-pressure	−0.35	0.11	0.01
		Unaltered	0.17	0.09	0.32
	Unaltered	Low-frequency	−0.22	0.09	0.11
		Low-frequency/low-pressure	−0.52	0.10	<0.01
		Low-pressure	−0.17	0.09	0.32
Rational	Low-frequency	Low-frequency/low-pressure	0.10	0.11	1.00
		Low-pressure	0.19	0.10	0.40
		Unaltered	0.07	0.12	1.00
	Low-frequency/low-pressure	Low-frequency	−0.10	0.11	1.00
		Low-pressure	0.09	0.11	1.00
		Unaltered	−0.03	0.11	1.00
	Low-pressure	Low-frequency	−0.19	0.10	0.40
		Low-frequency/low-pressure	−0.09	0.11	1.00
		Unaltered	−0.12	0.12	1.00
	Unaltered	Low-frequency	−0.07	0.12	1.00
		Low-frequency/low-pressure	0.03	0.11	1.00
		Low-pressure	0.12	0.12	1.00
Responsible	Low-frequency	Low-frequency/low-pressure	−0.10	0.11	1.00
		Low-pressure	−0.08	0.10	1.00
		Unaltered	0.18	0.09	0.36
	Low-frequency/low-pressure	Low-frequency	0.10	0.11	1.00
		Low-pressure	0.02	0.10	1.00
		Unaltered	0.28	0.10	0.04
	Low-pressure	Low-frequency	0.08	0.10	1.00
		Low-frequency/low-pressure	−0.02	0.10	1.00
		Unaltered	0.26	0.10	0.08
	Unaltered	Low-frequency	−0.18	0.09	0.36
		Low-frequency/low-pressure	−0.28	0.10	0.04
		Low-pressure	−0.26	0.10	0.08

**Table A2:** Summary of the results of the pairwise comparisons of Experiment 1 between-participants condition (sounds of packaging auditory experience) for emotion scores.

Measure	Stimuli		Mean Difference	Std. Error	*p*-Value
Amused	Bottle opening	Bottle pouring	−0.82	0.31	0.05
		Can opening	0.15	0.31	1.00
		Can pouring	−0.22	0.31	1.00
	Bottle pouring	Bottle opening	0.82	0.31	0.05
		Can opening	0.97	0.31	0.01
		Can pouring	0.59	0.31	0.33
	Can opening	Bottle opening	−0.15	0.31	1.00
		Bottle pouring	−0.97	0.31	0.01
		Can pouring	−0.38	0.31	1.00
	Can pouring	Bottle opening	0.22	0.31	1.00
		Bottle pouring	−0.59	0.31	0.33
		Can opening	0.38	0.31	1.00
Calmed	Bottle opening	Bottle pouring	−0.78	0.28	0.03
		Can opening	0.14	0.28	1.00
		Can pouring	−0.16	0.28	1.00
	Bottle pouring	Bottle opening	0.78	0.28	0.03
		Can opening	0.92	0.28	0.01
		Can pouring	0.62	0.28	0.16
	Can opening	Bottle opening	−0.14	0.28	1.00
		Bottle pouring	−0.92	0.28	0.01
		Can pouring	−0.30	0.28	1.00
	Can pouring	Bottle opening	0.16	0.28	1.00
		Bottle pouring	−0.62	0.28	0.16
		Can opening	0.30	0.28	1.00
Comforted	Bottle opening	Bottle pouring	−1.09	0.29	<0.01
		Can opening	−0.07	0.29	1.00
		Can pouring	−0.36	0.29	1.00
	Bottle pouring	Bottle opening	1.09	0.29	<0.01
		Can opening	1.02	0.29	<0.01
		Can pouring	0.72	0.29	0.07
	Can opening	Bottle opening	0.07	0.29	1.00
		Bottle pouring	−1.02	0.29	<0.01
		Can pouring	−0.30	0.29	1.00
	Can pouring	Bottle opening	0.36	0.29	1.00
		Bottle pouring	−0.72	0.29	0.07
		Can opening	0.30	0.29	1.00
Curious	Bottle opening	Bottle pouring	−0.64	0.31	0.24
		Can opening	0.07	0.31	1.00
		Can pouring	−0.52	0.31	0.56
	Bottle pouring	Bottle opening	0.64	0.31	0.24
		Can opening	0.71	0.31	0.13
		Can pouring	0.12	0.31	1.00
	Can opening	Bottle opening	−0.07	0.31	1.00
		Bottle pouring	−0.71	0.31	0.13
		Can pouring	−0.59	0.31	0.33
	Can pouring	Bottle opening	0.52	0.31	0.56
		Bottle pouring	−0.12	0.31	1.00
		Can opening	0.59	0.31	0.33
Disappointed	Bottle opening	Bottle pouring	0.39	0.25	0.72
		Can opening	−0.64	0.25	0.07
		Can pouring	−0.23	0.25	1.00
	Bottle pouring	Bottle opening	−0.39	0.25	0.72
		Can opening	−10.03	0.25	<0.01
		Can pouring	−0.62	0.25	0.08
	Can opening	Bottle opening	0.64	0.25	0.07
		Bottle pouring	10.03	0.25	<0.01
		Can pouring	0.41	0.25	0.61
	Can pouring	Bottle opening	0.23	0.25	1.00
		Bottle pouring	0.62	0.25	0.08
		Can opening	−0.41	0.25	0.61
Energetic	Bottle opening	Bottle pouring	−0.25	0.31	1.00
		Can opening	0.47	0.31	0.76
		Can pouring	0.16	0.31	1.00
	Bottle pouring	Bottle opening	0.25	0.31	1.00
		Can opening	0.72	0.31	0.12
		Can pouring	0.41	0.31	1.00
	Can opening	Bottle opening	−0.47	0.31	0.76
		Bottle pouring	−0.72	0.31	0.12
		Can pouring	−0.31	0.31	1.00
	Can pouring	Bottle opening	−0.16	0.31	1.00
		Bottle pouring	−0.41	0.31	1.00
		Can opening	0.31	0.31	1.00
Excited	Bottle opening	Bottle pouring	−0.74	0.31	0.11
		Can opening	0.37	0.31	1.00
		Can pouring	0.22	0.31	1.00
	Bottle pouring	Bottle opening	0.74	0.31	0.11
		Can opening	10.11	0.31	<0.01
		Can pouring	0.96	0.31	0.01
	Can opening	Bottle opening	−0.37	0.31	1.00
		Bottle pouring	−1.11	0.31	<0.01
		Can pouring	−0.15	0.31	1.00
	Can pouring	Bottle opening	−0.22	0.31	1.00
		Bottle pouring	−0.96	0.31	0.01
		Can opening	0.15	0.31	1.00
Friendly	Bottle opening	Bottle pouring	−0.77	0.32	0.09
		Can opening	0.35	0.32	1.00
		Can pouring	−0.05	0.32	1.00
	Bottle pouring	Bottle opening	0.77	0.32	0.09
		Can opening	1.12	0.32	<0.01
		Can pouring	0.72	0.32	0.14
	Can opening	Bottle opening	−0.35	0.32	1.00
		Bottle pouring	−1.12	0.32	<0.01
		Can pouring	−0.40	0.32	1.00
	Can pouring	Bottle opening	0.05	0.32	1.00
		Bottle pouring	−0.72	0.32	0.14
		Can opening	0.40	0.32	1.00
Good	Bottle opening	Bottle pouring	−0.74	0.27	0.04
		Can opening	0.50	0.27	0.39
		Can pouring	−0.02	0.27	1.00
	Bottle pouring	Bottle opening	0.74	0.27	0.04
		Can opening	1.24	0.27	<0.01
		Can pouring	0.72	0.27	0.05
	Can opening	Bottle opening	−0.50	0.27	0.39
		Bottle pouring	−1.24	0.27	<0.01
		Can pouring	−0.52	0.27	0.35
	Can pouring	Bottle opening	0.02	0.27	1.00
		Bottle pouring	−0.72	0.27	0.05
		Can opening	0.52	0.27	0.35
Grumpy	Bottle opening	Bottle pouring	0.30	0.22	1.00
		Can opening	−0.24	0.22	1.00
		Can pouring	−0.46	0.22	0.23
	Bottle pouring	Bottle opening	−0.30	0.22	1.00
		Can opening	−0.54	0.22	0.10
		Can pouring	−0.76	0.22	<0.01
	Can opening	Bottle opening	0.24	0.22	1.00
		Bottle pouring	0.54	0.22	0.10
		Can pouring	−0.22	0.22	1.00
	Can pouring	Bottle opening	0.46	0.22	0.23
		Bottle pouring	0.76	0.22	<0.01
		Can opening	0.22	0.22	1.00
Joyful	Bottle opening	Bottle pouring	−0.61	0.31	0.28
		Can opening	0.31	0.31	1.00
		Can pouring	0.08	0.31	1.00
	Bottle pouring	Bottle opening	0.61	0.31	0.28
		Can opening	0.92	0.31	0.02
		Can pouring	0.69	0.31	0.15
	Can opening	Bottle opening	−0.31	0.31	1.00
		Bottle pouring	−0.92	0.31	0.02
		Can pouring	−0.23	0.31	1.00
	Can pouring	Bottle opening	−0.08	0.31	1.00
		Bottle pouring	−0.69	0.31	0.15
		Can opening	0.23	0.31	1.00
Pleased	Bottle opening	Bottle pouring	−1.08	0.29	<0.01
		Can opening	0.22	0.29	1.00
		Can pouring	−0.15	0.29	1.00
	Bottle pouring	Bottle opening	1.08	0.29	<0.01
		Can opening	1.30	0.29	<0.01
		Can pouring	0.93	0.29	0.01
	Can opening	Bottle opening	−0.22	0.29	1.00
		Bottle pouring	−1.30	0.29	<0.01
		Can pouring	−0.37	0.29	1.00
	Can pouring	Bottle opening	0.15	0.29	1.00
		Bottle pouring	−0.93	0.29	0.01
		Can opening	0.37	0.29	1.00
Restless	Bottle opening	Bottle pouring	0.27	0.25	1.00
		Can opening	−0.27	0.25	1.00
		Can pouring	−0.61	0.25	0.09
	Bottle pouring	Bottle opening	−0.27	0.25	1.00
		Can opening	−0.54	0.25	0.20
		Can pouring	−0.89	0.25	<0.01
	Can opening	Bottle opening	0.27	0.25	1.00
		Bottle pouring	0.54	0.25	0.20
		Can pouring	−0.35	0.25	1.00
	Can pouring	Bottle opening	0.61	0.25	0.09
		Bottle pouring	0.89	0.25	<0.01
		Can opening	0.35	0.25	1.00
Sad	Bottle opening	Bottle pouring	0.24	0.20	1.00
		Can opening	−0.24	0.20	1.00
		Can pouring	−0.25	0.20	1.00
	Bottle pouring	Bottle opening	−0.24	0.20	1.00
		Can opening	−0.48	0.20	0.09
		Can pouring	−0.49	0.20	0.08
	Can opening	Bottle opening	0.24	0.20	1.00
		Bottle pouring	0.48	0.20	0.09
		Can pouring	−0.01	0.20	1.00
	Can pouring	Bottle opening	0.25	0.20	1.00
		Bottle pouring	0.49	0.20	0.08
		Can opening	0.01	0.20	1.00
Rational	Bottle opening	Bottle pouring	−0.52	0.28	0.37
		Can opening	−0.05	0.28	1.00
		Can pouring	−0.71	0.28	0.07
	Bottle pouring	Bottle opening	0.52	0.28	0.37
		Can opening	0.47	0.28	0.56
		Can pouring	−0.19	0.28	1.00
	Can opening	Bottle opening	0.05	0.28	1.00
		Bottle pouring	−0.47	0.28	0.56
		Can pouring	−0.66	0.28	0.12
	Can pouring	Bottle opening	0.71	0.28	0.07
		Bottle pouring	0.19	0.28	1.00
		Can opening	0.66	0.28	0.12
Responsible	Bottle opening	Bottle pouring	−0.49	0.29	0.53
		Can opening	−0.01	0.29	1.00
		Can pouring	−0.21	0.29	1.00
	Bottle pouring	Bottle opening	0.49	0.29	0.53
		Can opening	0.48	0.29	0.55
		Can pouring	0.28	0.29	1.00
	Can opening	Bottle opening	0.01	0.29	1.00
		Bottle pouring	−0.48	0.29	0.55
		Can pouring	−0.21	0.29	1.00
	Can pouring	Bottle opening	0.21	0.29	1.00
		Bottle pouring	−0.28	0.29	1.00
		Can opening	0.21	0.29	1.00

**Table A3:** Summary of the results of the pairwise comparisons of Experiment 1 within-participants condition (sound manipulation) for sensory expectations.

Measure	Stimuli		Mean Difference	Std. Error	*p*-Value
Alcohol	Low-frequency	Low-frequency/low-pressure	0.50	0.13	<0.01
		Low-pressure	0.56	0.13	<0.01
		Unaltered	0.08	0.10	1.00
	Low-frequency/low-pressure	Low-frequency	−0.50	0.13	<0.01
		Low-pressure	0.06	0.10	1.00
		Unaltered	−0.42	0.12	<0.01
	Low-pressure	Low-frequency	−0.56	0.13	<0.01
		Low-frequency/low-pressure	−0.06	0.10	1.00
		Unaltered	−0.48	0.11	<0.01
	Unaltered	Low-frequency	−0.08	0.10	1.00
		Low-frequency/low-pressure	0.42	0.12	<0.01
		Low-pressure	0.48	0.11	<0.01
Refreshing	Low-frequency	Low-frequency/low-pressure	1.00	0.17	<0.01
		Low-pressure	0.45	0.15	0.02
		Unaltered	−0.14	0.13	1.00
	Low-frequency/low-pressure	Low-frequency	−1.00	0.17	<0.01
		Low-pressure	−0.54	0.13	<0.01
		Unaltered	−1.13	0.16	<0.01
	Low-pressure	Low-frequency	−0.45	0.15	0.02
		Low-frequency/low-pressure	0.54	0.13	<0.01
		Unaltered	−0.59	0.15	<0.01
	Unaltered	Low-frequency	0.14	0.13	1.00
		Low-frequency/low-pressure	1.13	0.16	<0.01
		Low-pressure	0.59	0.15	<0.01
Sweetness	Low-frequency	Low-frequency/low-pressure	−0.12	0.13	1.00
		Low-pressure	−0.44	0.14	0.02
		Unaltered	−0.26	0.12	0.21
	Low-frequency/low-pressure	Low-frequency	0.12	0.13	1.00
		Low-pressure	−0.32	0.14	0.12
		Unaltered	−0.14	0.13	1.00
	Low-pressure	Low-frequency	0.44	0.14	0.02
		Low-frequency/low-pressure	0.32	0.14	0.12
		Unaltered	0.18	0.14	1.00
	Unaltered	Low-frequency	0.26	0.12	0.21
		Low-frequency/low-pressure	0.14	0.13	1.00
		Low-pressure	−0.18	0.14	1.00
Bitterness	Low-frequency	Low-frequency/low-pressure	0.20	0.14	1.00
		Low-pressure	0.49	0.15	0.01
		Unaltered	0.27	0.14	0.33
	Low-frequency/low-pressure	Low-frequency	−0.20	0.14	1.00
		Low-pressure	0.29	0.15	0.28
		Unaltered	0.07	0.15	1.00
	Low-pressure	Low-frequency	−0.49	0.15	0.01
		Low-frequency/low-pressure	−0.29	0.15	0.28
		Unaltered	−0.22	0.15	0.77
	Unaltered	Low-frequency	−0.27	0.14	0.33
		Low-frequency/low-pressure	−0.07	0.15	1.00
		Low-pressure	0.22	0.15	0.77

**Table A4:** Summary of the results of the pairwise comparisons of Experiment 1 between-participants condition (sounds of packaging auditory experience) for sensory expectations.

Measure	Stimuli		Mean Difference	Std. Error	*p*-Value
Alcohol	Bottle opening	Bottle pouring	0.07	0.33	1.00
		Can opening	0.39	0.33	1.00
		Can pouring	−0.12	0.33	1.00
	Bottle pouring	Bottle opening	−0.07	0.33	1.00
		Can opening	0.32	0.33	1.00
		Can pouring	−0.19	0.33	1.00
	Can opening	Bottle opening	−0.39	0.33	1.00
		Bottle pouring	−0.32	0.33	1.00
		Can pouring	−0.51	0.33	0.75
	Can pouring	Bottle opening	0.12	0.33	1.00
		Bottle pouring	0.19	0.33	1.00
		Can opening	0.51	0.33	0.75
Refreshing	Bottle opening	Bottle pouring	−1.09	0.28	<0.01
		Can opening	0.33	0.28	1.00
		Can pouring	−0.34	0.28	1.00
	Bottle pouring	Bottle opening	1.09	0.28	<0.01
		Can opening	1.42	0.28	<0.01
		Can pouring	0.75	0.28	0.05
	Can opening	Bottle opening	−0.33	0.28	1.00
		Bottle pouring	−1.42	0.28	<0.01
		Can pouring	−0.67	0.28	0.10
	Can pouring	Bottle opening	0.34	0.28	1.00
		Bottle pouring	−0.75	0.28	0.05
		Can opening	0.67	0.28	0.10
Sweetness	Bottle opening	Bottle pouring	−0.35	0.27	1.00
		Can opening	−0.27	0.27	1.00
		Can pouring	−0.69	0.27	0.06
	Bottle pouring	Bottle opening	0.35	0.27	1.00
		Can opening	0.08	0.27	1.00
		Can pouring	−0.34	0.27	1.00
	Can opening	Bottle opening	0.27	0.27	1.00
		Bottle pouring	−0.08	0.27	1.00
		Can pouring	−0.42	0.27	0.69
	Can pouring	Bottle opening	0.69	0.27	0.06
		Bottle pouring	0.34	0.27	1.00
		Can opening	0.42	0.27	0.69
Bitterness	Bottle opening	Bottle pouring	−0.20	0.30	1.00
		Can opening	−0.10	0.30	1.00
		Can pouring	−0.61	0.30	0.26
	Bottle pouring	Bottle opening	0.20	0.30	1.00
		Can opening	0.10	0.30	1.00
		Can pouring	−0.41	0.30	1.00
	Can opening	Bottle opening	0.10	0.30	1.00
		Bottle pouring	−0.10	0.30	1.00
		Can pouring	−0.51	0.30	0.56
	Can pouring	Bottle opening	0.61	0.30	0.26
		Bottle pouring	0.41	0.30	1.00
		Can opening	0.51	0.30	0.56

**Table A5:** Summary of the results of the pairwise comparisons of auditory conditions for emotion scores in Experiment 2.

Measure	Stimuli		Mean Difference	Std. Error	*p*-Value
Amused	No sound	Unaltered	−0.47	0.24	0.32
		Low-frequency	−0.22	0.24	1.00
		Low-pressure	−0.35	0.24	0.89
	Unaltered	No sound	0.47	0.24	0.32
		Low-frequency	0.25	0.24	1.00
		Low-pressure	0.12	0.24	1.00
	Low-frequency	No sound	0.22	0.24	1.00
		Unaltered	−0.25	0.24	1.00
		Low-pressure	−0.13	0.24	1.00
	Low-pressure	No sound	0.35	0.24	0.89
		Unaltered	−0.12	0.24	1.00
		Low-frequency	0.13	0.24	1.00
Calmed	No sound	Unaltered	−1.22	0.23	<0.01
		Low-frequency	−1.09	0.23	<0.01
		Low-pressure	−1.30	0.23	<0.01
	Unaltered	No sound	1.22	0.23	<0.01
		Low-frequency	0.12	0.23	1.00
		Low-pressure	−0.08	0.23	1.00
	Low-frequency	No sound	1.09	0.23	<0.01
		Unaltered	−0.12	0.23	1.00
		Low-pressure	−0.20	0.23	1.00
	Low-pressure	No sound	1.30	0.23	<0.01
		Unaltered	0.08	0.23	1.00
		Low-frequency	0.20	0.23	1.00
Comforted	No sound	Unaltered	−1.00	0.22	<0.01
		Low-frequency	−0.91	0.22	<0.01
		Low-pressure	−1.12	0.22	<0.01
	Unaltered	No sound	1.00	0.22	<0.01
		Low-frequency	0.09	0.22	1.00
		Low-pressure	−0.12	0.22	1.00
	Low-frequency	No sound	0.91	0.22	<0.01
		Unaltered	−0.09	0.22	1.00
		Low-pressure	−0.21	0.22	1.00
	Low-pressure	No sound	1.12	0.22	<0.01
		Unaltered	0.12	0.22	1.00
		Low-frequency	0.21	0.22	1.00
Curious	No sound	Unaltered	−0.10	0.24	1.00
		Low-frequency	−0.04	0.24	1.00
		Low-pressure	−0.13	0.24	1.00
	Unaltered	No sound	0.10	0.24	1.00
		Low-frequency	0.06	0.24	1.00
		Low-pressure	−0.03	0.24	1.00
	Low-frequency	No sound	0.04	0.24	1.00
		Unaltered	−0.06	0.24	1.00
		Low-pressure	−0.09	0.24	1.00
	Low-pressure	No sound	0.13	0.24	1.00
		Unaltered	0.03	0.24	1.00
		Low-frequency	0.09	0.24	1.00
Disappointed	No sound	Unaltered	0.72	0.18	<0.01
		Low-frequency	0.70	0.18	<0.01
		Low-pressure	0.75	0.18	<0.01
	Unaltered	No sound	−0.72	0.18	<0.01
		Low-frequency	−0.01	0.18	1.00
		Low-pressure	0.03	0.18	1.00
	Low-frequency	No sound	−0.70	0.18	<0.01
		Unaltered	0.01	0.18	1.00
		Low-pressure	0.05	0.18	1.00
	Low-pressure	No sound	−0.75	0.18	<0.01
		Unaltered	−0.03	0.18	1.00
		Low-frequency	−0.05	0.18	1.00
Energetic	No sound	Unaltered	−0.26	0.22	1.00
		Low-frequency	−0.48	0.22	0.17
		Low-pressure	−0.23	0.22	1.00
	Unaltered	No sound	0.26	0.22	1.00
		Low-frequency	−0.22	0.22	1.00
		Low-pressure	0.03	0.22	1.00
	Low-frequency	No sound	0.48	0.22	0.17
		Unaltered	0.22	0.22	1.00
		Low-pressure	0.25	0.22	1.00
	Low-pressure	No sound	0.23	0.22	1.00
		Unaltered	−0.03	0.22	1.00
		Low-frequency	−0.25	0.22	1.00
Excited	No sound	Unaltered	−0.80	0.23	<0.01
		Low-frequency	−0.46	0.23	0.29
		Low-pressure	−0.79	0.23	<0.01
	Unaltered	No sound	0.80	0.23	<0.01
		Low-frequency	0.34	0.23	0.86
		Low-pressure	0.01	0.23	1.00
	Low-frequency	No sound	0.46	0.23	0.29
		Unaltered	−0.34	0.23	0.86
		Low-pressure	−0.33	0.23	0.93
	Low-pressure	No sound	0.79	0.23	<0.01
		Unaltered	−0.01	0.23	1.00
		Low-frequency	0.33	0.23	0.93
Friendly	No sound	Unaltered	−0.42	0.21	0.30
		Low-frequency	−0.41	0.21	0.34
		Low-pressure	−0.61	0.21	0.03
	Unaltered	No sound	0.42	0.21	0.30
		Low-frequency	0.01	0.21	1.00
		Low-pressure	−0.20	0.21	1.00
	Low-frequency	No sound	0.41	0.21	0.34
		Unaltered	−0.01	0.21	1.00
		Low-pressure	−0.20	0.21	1.00
	Low-pressure	No sound	0.61	0.21	0.03
		Unaltered	0.20	0.21	1.00
		Low-frequency	0.20	0.21	1.00
Good	No sound	Unaltered	−0.76	0.20	<0.01
		Low-frequency	−0.62	0.20	0.01
		Low-pressure	−0.65	0.20	0.01
	Unaltered	No sound	0.76	0.20	<0.01
		Low-frequency	0.15	0.20	1.00
		Low-pressure	0.11	0.20	1.00
	Low-frequency	No sound	0.62	0.20	0.01
		Unaltered	−0.15	0.20	1.00
		Low-pressure	−0.04	0.20	1.00
	Low-pressure	No sound	0.65	0.20	0.01
		Unaltered	−0.11	0.20	1.00
		Low-frequency	0.04	0.20	1.00
Grumpy	No sound	Unaltered	0.81	0.19	<0.01
		Low-frequency	0.41	0.19	0.18
		Low-pressure	0.52	0.19	0.04
	Unaltered	No sound	−0.81	0.19	<0.01
		Low-frequency	−0.40	0.19	0.21
		Low-pressure	−0.29	0.19	0.76
	Low-frequency	No sound	−0.41	0.19	0.18
		Unaltered	0.40	0.19	0.21
		Low-pressure	0.11	0.19	1.00
	Low-pressure	No sound	−0.52	0.19	0.04
		Unaltered	0.29	0.19	0.76
		Low-frequency	−0.11	0.19	1.00
Joyful	No sound	Unaltered	−0.75	0.22	<0.01
		Low-frequency	−0.74	0.22	0.01
		Low-pressure	−0.87	0.22	<0.01
	Unaltered	No sound	0.75	0.22	<0.01
		Low-frequency	0.01	0.22	1.00
		Low-pressure	−0.12	0.22	1.00
	Low-frequency	No sound	0.74	0.22	0.01
		Unaltered	−0.01	0.22	1.00
		Low-pressure	−0.13	0.22	1.00
	Low-pressure	No sound	0.87	0.22	<0.01
		Unaltered	0.12	0.22	1.00
		Low-frequency	0.13	0.22	1.00
Pleased	No sound	Unaltered	−1.11	0.21	<0.01
		Low-frequency	−0.82	0.21	<0.01
		Low-pressure	−0.94	0.21	<0.01
	Unaltered	No sound	1.11	0.21	<0.01
		Low-frequency	0.30	0.21	0.90
		Low-pressure	0.18	0.21	1.00
	Low-frequency	No sound	0.82	0.21	<0.01
		Unaltered	−0.30	0.21	0.90
		Low-pressure	−0.12	0.21	1.00
	Low-pressure	No sound	0.94	0.21	<0.01
		Unaltered	−0.18	0.21	1.00
		Low-frequency	0.12	0.21	1.00
Restless	No sound	Unaltered	0.42	0.21	0.29
		Low-frequency	0.37	0.21	0.50
		Low-pressure	0.44	0.21	0.23
	Unaltered	No sound	−0.42	0.21	0.29
		Low-frequency	−0.05	0.21	1.00
		Low-pressure	0.02	0.21	1.00
	Low-frequency	No sound	−0.37	0.21	0.50
		Unaltered	0.05	0.21	1.00
		Low-pressure	0.07	0.21	1.00
	Low-pressure	No sound	−0.44	0.21	0.23
		Unaltered	−0.02	0.21	1.00
		Low-frequency	−0.07	0.21	1.00
Sad	No sound	Unaltered	0.60	0.18	<0.01
		Low-frequency	0.44	0.18	0.08
		Low-pressure	0.38	0.18	0.19
	Unaltered	No sound	−0.60	0.18	<0.01
		Low-frequency	−0.16	0.18	1.00
		Low-pressure	−0.22	0.18	1.00
	Low-frequency	No sound	−0.44	0.18	0.08
		Unaltered	0.16	0.18	1.00
		Low-pressure	−0.06	0.18	1.00
	Low-pressure	No sound	−0.38	0.18	0.19
		Unaltered	0.22	0.18	1.00
		Low-frequency	0.06	0.18	1.00
Rational	No sound	Unaltered	0.10	0.22	1.00
		Low-frequency	0.21	0.22	1.00
		Low-pressure	−0.19	0.22	1.00
	Unaltered	No sound	−0.10	0.22	1.00
		Low-frequency	0.12	0.22	1.00
		Low-pressure	−0.29	0.22	1.00
	Low-frequency	No sound	−0.21	0.22	1.00
		Unaltered	−0.12	0.22	1.00
		Low-pressure	−0.41	0.22	0.38
	Low-pressure	No sound	0.19	0.22	1.00
		Unaltered	0.29	0.22	1.00
		Low-frequency	0.41	0.22	0.38
Responsible	No sound	Unaltered	0.65	0.23	0.03
		Low-frequency	0.79	0.24	0.01
		Low-pressure	0.14	0.24	1.00
	Unaltered	No sound	−0.65	0.23	0.03
		Low-frequency	0.14	0.24	1.00
		Low-pressure	−0.51	0.23	0.17
	Low-frequency	No sound	−0.79	0.24	0.01
		Unaltered	−0.14	0.24	1.00
		Low-pressure	−0.65	0.24	0.04
	Low-pressure	No sound	−0.14	0.24	1.00
		Unaltered	0.51	0.23	0.17
		Low-frequency	0.65	0.24	0.04

**Table A6:** Summary of the results of the pairwise comparisons of auditory conditions on each sensory expectation in Experiment 2.

Measure	Stimuli		Mean Difference	Std. Error	*p*-Value
Refreshing	No sound	Unaltered	−0.66	0.17	<0.01
		Low-frequency	−0.43	0.18	0.09
		Low-pressure	−0.64	0.18	<0.01
	Unaltered	No sound	0.66	0.17	<0.01
		Low-frequency	0.23	0.18	1.00
		Low-pressure	0.02	0.17	1.00
	Low-frequency	No sound	0.43	0.18	0.09
		Unaltered	−0.23	0.18	1.00
		Low-pressure	−0.21	0.18	1.00
	Low-pressure	No sound	0.64	0.18	<0.01
		Unaltered	−0.02	0.17	1.00
		Low-frequency	0.21	0.18	1.00
Sweetness	No sound	Unaltered	−0.27	0.23	1.00
		Low-frequency	−0.19	0.23	1.00
		Low-pressure	−0.36	0.23	0.74
	Unaltered	No sound	0.27	0.23	1.00
		Low-frequency	0.07	0.23	1.00
		Low-pressure	−0.09	0.23	1.00
	Low-frequency	No sound	0.19	0.23	1.00
		Unaltered	−0.07	0.23	1.00
		Low-pressure	−0.16	0.23	1.00
	Low-pressure	No sound	0.36	0.23	0.74
		Unaltered	0.09	0.23	1.00
		Low-frequency	0.16	0.23	1.00
Bitterness	No sound	Unaltered	0.20	0.24	1.00
		Low-frequency	0.57	0.25	0.13
		Low-pressure	0.22	0.25	1.00
	Unaltered	No sound	−0.20	0.24	1.00
		Low-frequency	0.37	0.24	0.78
		Low-pressure	0.02	0.24	1.00
	Low-frequency	No sound	−0.57	0.25	0.13
		Unaltered	−0.37	0.24	0.78
		Low-pressure	−0.35	0.24	0.91
	Low-pressure	No sound	−0.22	0.25	1.00
		Unaltered	−0.02	0.24	1.00
		Low-frequency	0.35	0.24	0.91
Refreshing	No sound	Unaltered	−0.66	0.17	<0.01
		Low-frequency	−0.43	0.18	0.09
		Low-pressure	−0.64	0.18	<0.01
	Unaltered	No sound	0.66	0.17	<0.01
		Low-frequency	0.23	0.18	1.00
		Low-pressure	0.02	0.17	1.00
	Low-frequency	No sound	0.43	0.18	0.09
		Unaltered	−0.23	0.18	1.00
		Low-pressure	−0.21	0.18	1.00
	Low-pressure	No sound	0.64	0.18	<0.01
		Unaltered	−0.02	0.17	1.00
		Low-frequency	0.21	0.18	1.00
